# External Root Resorption Associated with Orthodontic Treatment—Descriptive Correlations of Biological and Dental Risk Factors

**DOI:** 10.3390/dj14010042

**Published:** 2026-01-07

**Authors:** Maria-Cristina Zlate, Maria-Angelica Bencze, Anca-Oana Dragomirescu, Andreea-Mihaela Bǎluțǎ, Ecaterina Ionescu

**Affiliations:** 1 Department of Orthodontics and Dentofacial Orthopaedics, Faculty of Dental Medicine, “Carol Davila” University of Medicine and Pharmacy, 020021 Bucharest, Romania; maria-cristina.ionica@drd.umfcd.ro (M.-C.Z.); maria.bencze@umfcd.ro (M.-A.B.); ecaterina.ionescu@umfcd.ro (E.I.); 2 Department of Esthetics, Faculty of Dental Medicine, “Carol Davila” University of Medicine and Pharmacy, 020021 Bucharest, Romania; andreea.baluta@umfcd.ro

**Keywords:** root resorption, orthodontic treatment, risk factors, age, gender, facial pattern, dento-maxillary anomaly

## Abstract

**Background/Objectives**: External root resorption is an undesirable complication of orthodontic treatment, characterized by the loss of dental root structure. The aim of this study was to identify the biological and dental risk factors involved in the development of external root resorption at the end of orthodontic treatment. **Methods**: A retrospective observational study was conducted on a sample of 120 patients who underwent orthodontic treatment. External root resorption was assessed using pre- and post-treatment panoramic radiographs. Correlations were established between the severity of external root resorption and various biological and dental risk factors. **Results**: Out of a total of 2639 teeth analyzed, 52.14% exhibited external root resorption, with most cases being mild to moderate (<3 mm). The maxillary central incisors were the most affected teeth. Age showed a statistically significant correlation with the severity of external root resorption in the lower anterior region (*p* < 0.01). No significant differences were observed in relation to gender, facial growth pattern, or type of dento-maxillary anomaly. **Conclusions**: External root resorption is a common consequence of orthodontic treatment, most often presenting with low severity. The type of tooth and the patient’s age influence the severity of root resorption, while factors such as gender, facial growth pattern, and type of dento-maxillary anomaly did not prove to be significant in this context.

## 1. Introduction

Root resorption is defined as the progressive loss of cementum or dentin at the root level due to osteoclastic activity. According to most authors, it represents an undesirable but frequently encountered side effect of orthodontic treatment [1,2].

The most common form of root resorption resulting from orthodontic treatment is external root resorption, which leads to the permanent loss of root dental structure, typically starting at the apex [2].

Orthodontic treatment is not the sole factor responsible for the onset of external root resorption [2,3], but data from the literature indicate that patients undergoing orthodontic treatment are more prone to developing severe forms of external root resorption. Therefore, it is important for orthodontic specialists to identify risk factors for external root resorption prior to initiating orthodontic therapy [4].

Multiple factors, broadly classified into patient-related and treatment-related factors, have been implicated in the onset and progression of external root resorption [5].

Patient-related risk factors include biological characteristics such as age, gender [6], facial pattern, genetic susceptibility [7], systemic factors such as chronic asthma and hormonal disorders [8] and others. Local dental factors also play a crucial role. These include tooth predisposition [9], type and severity of malocclusion [10], parafunctional habits—such as bruxism, tongue thrusting, finger sucking, and nail-biting [11] and a history of dental trauma, which may predispose the root to structural compromise [11].

The treatment-related factors include duration of treatment [12], direction and magnitude of force [11], type of appliance and a history of tooth extraction [13], treatment mechanics and others. These factors can interact with each other and result in the development of external root resorption [5].

In this context, the aim of the present study was to identify biological and dental risk factors associated with external root resorption observed at the end of orthodontic treatment.

## 2. Materials and Methods

To determine whether there are correlations between external root resorption and certain patient characteristics, as possible risk factors of external root resorption in the context of orthodontic treatment, we conducted a retrospective observational descriptive study.

The cases were selected from patients who received orthodontic treatment with either fixed or removable appliances at the Department of Orthodontics and Dentofacial Orthopaedics, Faculty of Dental Medicine, “Carol Davila” University of Medicine and Pharmacy, Bucharest, within the External Clinical Section of the Dental Outpatient Clinic of The University Emergency Hospital Bucharest.

Inclusion criteria were:

- Complete orthodontic records, including two orthopantomograms (one initial and one at the end of treatment);
- Orthodontic treatment on both arches;
- Age at the start of treatment over 12 years (root formation completed).

Exclusion criteria included:

- Systemic diseases affecting dental tissues (asthma, allergies, hormonal imbalances, skeletal metabolic disorders);
- Local conditions that could influence root resorption (periapical infections, maxillary tumors or cysts);
- History of dento-maxillary trauma;
- Previous orthodontic treatment;
- External root resorption present before starting orthodontic treatment;
- Cases where root apices cannot be correctly visualized on radiographs;
- Changes in tooth length during treatment due to dental procedures.

Based on the inclusion and exclusion criteria, the final study group consisted of 120 patients. For each patient, data were recorded regarding the age at the start of orthodontic treatment, gender, facial pattern, and type of dento-maxillary anomaly.

To determine the facial pattern, we analyzed the angle formed between the mandibular plane and the Frankfort horizontal plane (FMA), with the following classifications: normodivergent facial pattern for angles between 22° and 28°, hypodivergent for angles below 22°, and hyperdivergent for angles above 28°.

Dento-maxillary anomalies were assessed using Angle’s classification into three classes [14]: class I—dento-maxillary anomalies with a neutral occlusal relationship of the first permanent molars, class II—dento-maxillary anomalies with a bilateral distal occlusal relationship of the first permanent molars in the sagittal plane, associated with protruded upper incisors (Class II/1 anomaly), or retroclined upper incisors (Class II/2 anomaly), class III—dento-maxillary anomalies with a mesial occlusal relationship of the first permanent molars in the sagittal plane.

To evaluate the presence and severity of external root resorption, panoramic radiographs taken at the beginning and at the end of orthodontic treatment were examined. We looked for changes in root contour (irregular outline, blunted shape) and reduction in root length.

Severity of external root resorption was quantified in millimeters using Linge’s formula [15]: R = (R1 − R2) × C1/C2 (where R = root resorption, R1 = root length before treatment, R2 = root length after treatment, C1 = crown length before treatment, C2 = crown length after treatment).

To ensure reproductible and accurate measurements, the anatomical crown was distinguished from the root by using the midpoint of the line connecting the mesial and distal cemento-enamel junctions of each tooth [16].

We calculated the mean resorption for each patient in four regions: upper anterior (upper incisors and canines), upper posterior (first and second premolars, and first molars), lower anterior (lower incisors and canines), lower posterior (first and second premolars, and first molars), using the formula: mean external root resorption = sum of resorption values/number of affected teeth.

Measurements were performed using the Webceph digital platform, after calibrating each radiograph. All measurements were recorded by a single examiner. To assess measurement reliability, 30 randomly selected panoramic X-rays were re-analyzed by the same examiner. The mean intra-observer ICC was 0.968, with a 95% confidence interval of 0.935–0.987, indicating an excellent level of agreement between repeated measurements by the same observer.

Data analysis was performed using IBM SPSS Statistics (version 26) and R Studio (version 2025.09.2 Build 418). The distribution of continuous variables was tested using the Kolmogorov–Smirnov test and graphical methods to determine the appropriateness of parametric or non-parametric tests. Qualitative variables were reported as frequencies and percentages, whereas quantitative variables were summarized using the mean and standard deviation for normally distributed data, or the median and interquartile range for non-normally distributed data. To evaluate the relationships between various factors and external root resorption, the Spearman correlation coefficient was used. A *p*-value < 0.05 was considered statistically significant.

The ethical approval was obtained for the study from the Research Ethics Committee of “Carol Davila” University of Medicine and Pharmacy, with the code PO-35-F-03.

## 3. Results

### 3.1. Characteristics of the Study Group

The age of patients at the start of orthodontic treatment ranged between 12 and 31 years, with a median age of 15 years. The majority of participants—86 patients (71.7%)—began orthodontic treatment before the age of 18, indicating a predominance of adolescents in the study.

From the perspective of patient distribution by gender, female patients predominated—92 patients, representing 76.67% of the total patients, while male patients—28 patients—accounted for only 23.33% of the sample.

Regarding facial pattern, the most frequently identified was the normodivergent facial pattern—45 patients (37.5%), followed by the hypodivergent—41 patients (34.2%), and the hyperdivergent—34 patients (28.3%).

The analysis of patient distribution according to the type of dento-maxillary anomaly (based on Angle’s classification) indicated a predominance of Class I anomalies—47 patients (39.2%), followed by Class II/2 dento-maxillary anomalies—34 patients (28.3%), and Class II/1 anomalies—21 patients (17.5%), while Class III anomaly was less frequent, found in only 18 patients (15.0%).

### 3.2. Severity of External Root Resorption

The overall distribution analysis of external root resorption shows that out of 2639 teeth analyzed, 1376 teeth (52.14%) were affected by external root resorption.

The Kolmogorov–Smirnov test proved that the distribution of continuous variables was non-normal, therefore non-parametric tests were used for appropriate inferential analysis.

Regarding the severity of external root resorption, most cases in the current analysis fell within the limits of mild to moderate resorption (Table 1). The most common were external root resorptions under 1 mm, representing 15.84% (418 teeth) of all teeth analyzed. As resorption severity increased, its frequency progressively decreased. These results suggest that, in most cases, external root resorption was limited in severity, and advanced cases were rare.

### 3.3. External Root Resorption by Tooth Type

Comparing external root resorption by tooth type (Table 2) showed significant differences between groups analyzed (*p* = 0.002). The highest mean values of external root resorption were recorded in maxillary central incisors (2.10 mm) and lateral incisors (2.02 mm), while the upper premolars had the lowest mean values of external root resorption (1.53 mm). The post hoc analysis with Bonferroni correction showed that only the maxillary central incisors and the maxillary lateral incisors had significantly more severe resorption than the maxillary premolars, while the other pairwise comparisons did not reach the threshold for statistical significance. Overall, these results support the idea that the maxillary incisors are the most vulnerable to root resorption, whereas the maxillary premolars exhibit the least impairment.

To simultaneously evaluate the influence of patient characteristics and tooth type on the severity of root resorption, a linear regression model (Table 3) was constructed with the tooth-level resorption score as the dependent variable, and age, gender, facial pattern, and tooth group as predictors. The model was significant overall (*p* < 0.001) but explained only 2.6% of the variability in the score (R^2^ = 0.026), indicating a modest overall predictive ability.

Among the included variables, age and tooth type showed independent associations with the severity of resorption. Each additional year of age was associated with a small but significant increase in the resorption score (*p* = 0.017), suggesting slightly more pronounced resorption in older patients. Using maxillary premolars as the reference group, most other tooth groups showed greater resorption. The most pronounced differences were observed in the maxillary central incisors (*p* < 0.001) and maxillary lateral incisors (*p* < 0.001), followed by the mandibular central incisors (*p* = 0.005) and mandibular lateral incisors (*p* = 0.004). Maxillary canines also showed significantly greater resorption compared to maxillary premolars (*p* = 0.017), while maxillary and mandibular molars, as well as mandibular canines, showed only trends toward higher values, near the threshold of statistical significance. Mandibular premolars did not differ significantly from maxillary premolars (*p* = 0.301). Gender and facial pattern (hypo- or hyperdivergent vs. normal) were not significantly associated with resorption severity after adjusting for the other factors.

The multivariate analysis confirms that, after controlling for age, gender, and facial pattern, the incisors—particularly the maxillary incisors—display the greatest root resorption compared with maxillary premolars, whereas general patient characteristics play a much smaller role.

### 3.4. External Root Resorption by Age and Gender

Age did not show significant correlations with the mean value of overall external root resorption for all teeth, but it was positively correlated with the mean resorption in the lower anterior area (r = 0.250, *p* < 0.01), suggesting that older patients tend to present more severe resorption in this region (Table 4).

When comparing by gender (Table 5), the values of average external root resorption overall and by region showed no significant differences between males and females.

### 3.5. External Root Resorption According to Facial Pattern

In this study, the analysis based on facial pattern did not reveal significant differences in the mean values of external root resorption (Table 6). The mean values were similar both in the upper anterior region (1.92 mm in patients with a hypodivergent facial pattern, 1.86 mm in those with a normodivergent pattern, and 1.85 mm in patients with a hyperdivergent pattern, *p* = 0.925), as well as in the upper posterior region (1.51 mm in hypodivergent patients, 1.55 mm in those with a normodivergent pattern, and 1.46 mm in hyperdivergent patients, *p* = 0.881). No significant differences were observed in the lower regions either, with values being similar across the three groups.

### 3.6. Correlation Between External Root Resorption and Type of Dento-Maxillary Anomaly

We compared the mean values of external root resorption according to the type of dento-maxillary anomaly (Angle’s classification), but no significant differences were found among the groups (Table 7). Our results suggest that the type of dento-maxillary anomaly treated orthodontically does not directly influence the development of external root resorption.

## 4. Discussion

External root resorption associated with orthodontic treatment is a relatively common complication that has posed, and continues to pose, a challenge for orthodontic specialists.

### 4.1. Prevalence and Severity of External Root Resorption

In the current analysis, out of a total of 2639 examined teeth, 1376 teeth (52.14%) were affected by external root resorption. Of the affected teeth, 1167 teeth—84.81%—showed resorption values of up to 3 mm. Severe resorption, exceeding 4 mm, was found in 56 teeth—4.07% of those affected by external root resorption.

Studies by Makedonas et al. [4] and Lund et al. [17], which investigated the prevalence of external root resorption associated with orthodontic treatment using computed tomography, showed that nearly all patients and up to 91% of teeth presented some degree of root shortening.

Research by Linge [15], Elhaddaoui et al. [18], Sameshima and Sinclair [19] demonstrated that when assessed using orthopantomograms or periapical radiographs, external root resorption does not exceed 2.5 mm and is classified as mild to moderate, with limited clinical relevance. In rare cases, severe external root resorption—exceeding 4 mm and considered equivalent to the loss of more than one-third of the original root length—was reported by Janson et al. [20], Weltman et al. [3], and Tieu et al. [21] in 1% to 10% of teeth treated orthodontically. Each study classified external root resorption differently; however, all concluded that most teeth exhibited mild to moderate resorption following treatment—similar to the conclusion reached in the present study.

Severe root resorption can significantly impair tooth function and reduce longevity; therefore, diligent radiographic monitoring during orthodontic treatment is crucial to promptly diagnose and manage external apical root resorption [22]. Considering that, in addition to local factors, systemic factors such as endocrine imbalances, metabolic disorders, co-infections and inflammatory processes can modify host responses and modulate susceptibility to root resorption, a coordinated approach encompassing both dental specialists and medical practitioners facilitates comprehensive risk assessment and optimized patient management [23,24]. Preventive strategies and interdisciplinary collaboration among healthcare professionals play a vital role in maintaining oral health and reducing the impact of treatment-related complications [25,26,27].

### 4.2. Age and Gender

Regarding age, our study did not find significant correlations with the mean value of external root resorption for all analyzed teeth. However, a positive correlation was observed with the mean resorption values for teeth in the lower anterior region, suggesting that older patients tend to exhibit more severe resorption in this area. This conclusion was also reached by Sameshima and Sinclair [19], who found that mandibular anterior teeth exhibit significant resorption when treatment is performed in adults.

Picanco et al. [6] observed that older patients have a higher risk of developing moderate or severe resorption compared to younger patients. This finding was explained by age-related changes in the periodontium: decreased vascularization, narrowing of the periodontal space, and thickening of the cementum [24,28]. However, most studies found that the age at the start of orthodontic treatment and the incidence of root resorption are only weakly correlated [21,29]. Genetic predispositions may also contribute to susceptibility to external root resorption. For instance, polymorphisms in genes related to inflammatory responses have been associated with periodontal disease and dento-maxillary anomalies, which share pathogenic mechanisms with orthodontically induced resorption [7,30].

When comparing mean root resorption values between genders for all teeth and by region, we did not find significant differences between female and male patients in the current study, with similar values of root shortening observed. This finding aligns with several studies in the literature, suggesting that gender is an unlikely risk factor for external root resorption [10,15,31]. In contrast, studies by Baghaei et al. [32] and Kjaer [33] indicated that female patients are more predisposed to external root resorption, while Bayir and Gumus [34] provided evidence that male patients are more predisposed.

### 4.3. Type of Dento-Maxillary Anomaly

In this study, external root resorption values were similar across all classes of dento-maxillary anomalies (based on Angle’s classification). Sondeijker et al. [35], in their recent clinical practice guideline, also concluded that there is no correlation between external root resorption and the type of dento-maxillary anomaly, but the quality of evidence was low.

According to the analysis by Baghaei et al. [32], patients with Angle Class I anomalies at the beginning of orthodontic treatment are less affected by severe external root resorption at the end of treatment. This is explained by the smaller movements undergone by the teeth—particularly the maxillary incisors. Regarding Class II and Class III anomalies, the authors reported a higher prevalence of severe resorption in the maxillary incisors among patients with Class III malocclusion. This finding may be due to the pressure exerted during treatment on the roots of the upper incisors in contact with the palatal cortical bone to compensate for the anomaly by tipping these teeth forward (vestibular inclination).

Tieu et al. [21] found limited evidence suggesting that treatment of Class II/1 malocclusion—regardless of the orthodontic strategy used—increases the severity and prevalence of external root resorption.

### 4.4. Facial Pattern

Vertical skeletal pattern assessment remains method-dependent. Angular parameters based on the Frankfort plane, such as the Frankfort–mandibular plane angle (FMA), as well as those based on the Sella–Nasion (SN) plane, such as the Sella–Nasion to mandibular plane angle (SN–MP), the Sella–Nasion to Gonion–Gnathion angle (SN–GoGn) and the Y-axis of growth (SNGn) are widely used in contemporary cephalometric analyses. FMA was selected in our study as the primary vertical descriptor due to its clinical interpretability and consistent landmark identification across the dataset.

In diagnostic performance analyses, FMA and SN–GoGn have repeatedly emerged as the most accurate indicators of facial vertical divergence, with FMA demonstrating high sensitivity and positive predictive values, particularly in normodivergent and hypodivergent subjects [36]. Furthermore, larger comparative investigations have shown FMA to be among the most reliable cephalometric indicators for vertical growth assessment, supporting the continued use of FMA when its selection is appropriately justified [37,38]. Comparative studies evaluating multiple vertical parameters consistently report similar classification success rates for SN-based measures and the Frankfort–mandibular plane angle (FMA), supporting the view that no single angular parameter is universally superior and that the validity of a given measure depends on landmark reproducibility and study design [36,37,39,40].

Although SN-referenced measures such as SN–GoGn and SN–MP are frequently considered reliable due to the relative stability of the cranial base, recent evidence indicates that these measurements are also influenced by anatomical factors including anterior cranial base length and inclination or due to Gn point shifting throughout sagittal discrepancies, which may affect their reproducibility and diagnostic performance [39,41,42]. Importantly, agreement among vertical cephalometric methods is moderate, and substantial concordance is typically observed only between specific parameter pairs, indicating that these measures are not fully interchangeable and that reliance on a single, well-defined descriptor may be appropriate when landmark visibility and reproducibility are prioritized [43].

In the current study, the analysis based on facial pattern did not reveal significant differences in the mean values of external root resorption. Similar results were obtained by Picanco et al. [6], who found no relationship between vertical growth pattern and the severity of root resorption.

In contrast, studies by Fernandes et al. [44] and Scheibel et al. [45] indicated that patients with a greater facial divergence (hyperdivergent facial pattern) may have an increased risk of significant external root resorption compared to those with lower divergence (hypodivergent facial pattern).

### 4.5. Affected Tooth

When comparing the mean external root resorption values by tooth type, our study revealed significant differences. The results suggest that the upper incisors are more prone to resorption than other teeth. The highest mean resorption values were recorded in the upper central incisors, followed in descending order by upper lateral incisors, lower central incisors, lower lateral incisors, maxillary canines, lower first molars, upper first molars, mandibular canines, mandibular premolars, while the upper premolars were the least affected.

In agreement with our findings on the susceptibility of certain teeth to external root resorption, evidence consistently highlights the maxillary incisors as the most susceptible, both in terms of prevalence and severity [3,22,46]. The higher risk of resorption in maxillary incisors during orthodontic treatment may be due to the greater distance these teeth typically move, the tipping/intrusion forces they are often subjected to compared to other teeth, and their smaller root surface area [46].

Most published studies reported that among the upper incisors, the central incisors are more susceptible to this process [46,47,48]—a conclusion also reached in our study.

On the other hand, Sameshima and Sinclair [49] found that the maxillary lateral incisors are more severely affected than the central incisors. According to them, after incisors, the most susceptible teeth in the upper arch to external root resorption are the first molars and canines.

In the lower arch, our study found that the incisors were most severely affected, followed by the first molar and the canine—a result also reported by Janson et al. [20]. This finding is not consistent with Sameshima and Sinclair’s study [49], which reported greater resorption in the mandibular canine than in any other mandibular tooth.

## 5. Study Limitations

This retrospective study has several limitations that should be considered when interpreting the findings. First, two-dimensional digital panoramic radiographs were used to assess root resorption; although widely available in clinical practice, they offer lower sensitivity compared with three-dimensional imaging techniques. Second, treatment-related variables such as appliance type, biomechanics, and treatment duration were not included in the analysis. These factors are inherently multifactorial and require a dedicated and more comprehensive analytical approach, which we plan to address in a future investigation. Finally, the gender distribution within the sample was markedly unbalanced, limiting the reliability of comparisons between male and female participants and indicating that any observed differences should be interpreted with caution.

## 6. Conclusions

In the present study, we found that over half of the analyzed teeth (52.14%) exhibited external root resorption by the end of orthodontic treatment. However, for 84.81% of the affected teeth, the severity was mild to moderate, remaining below 3 mm. Severe resorptions exceeding 5 mm were extremely rare, identified in only 1.16% of affected teeth. Although external apical root resorption was relatively common, most cases were mild to moderate, with severe resorption being rare, providing reassurance to clinicians and patients regarding the safety of routine orthodontic forces.

The study revealed that the upper central incisors were most prone to significant external root resorption, followed by the upper lateral incisors and the lower incisors. This suggests that the forces applied in the anterior arch regions have a greater impact on root integrity, highlighting the importance of careful force management in the anterior region and supporting the use of targeted radiographic monitoring.

For our study group, age was positively correlated with the mean external root resorption values for the anterior mandibular teeth, with older patients tending to exhibit more severe resorption in this region. This finding recommends closer monitoring and, if necessary, modified treatment protocols for older patients to minimize the risk of external root resorption during orthodontic treatment.

As for patient gender and facial pattern, these did not significantly influence the severity of external root resorption in the present study. The mean resorption values were similar regardless of the class of dento-maxillary anomaly.

## Figures and Tables

**Table 1:** Distribution of affected teeth by severity of external root resorption.

Severity of External Root Resorption	Number of Affected Teeth	Percentage of Total Teeth (n = 2639)	Percentage of Affected Teeth (n = 1376)
0–1 mm	418	15.84%	30.38%
1–2 mm	400	15.16%	29.07%
2–3 mm	349	13.22%	25.36%
3–4 mm	153	5.80%	11.12%
4–5 mm	40	1.52%	2.91%
5–6 mm	9	0.34%	0.65%
6–7 mm	5	0.19%	0.36%
7–8 mm	2	0.08%	0.15%

**Table 2:** External root resorption by tooth group.

	External Root Resorption (mm)				*p*
	Mean	SD	Median	IQR	
Maxillary central incisors	2.10	(1.32)	1.84	(1.07–2.67) ^a^	0.002 *
Mandibular central incisors	1.92	(1.18)	2.02	(0.85–2.65) ^ab^	
Maxillary lateral incisors	2.02	(1.24)	2.07	(0.77–2.63) ^a^	
Mandibular lateral incisors	1.91	(1.38)	1.80	(0.87–2.99) ^ab^	
Maxillary canines	1.84	(1.03)	1.89	(0.87–2.77) ^ab^	
Mandibular canines	1.76	(1.10)	1.57	(0.87–2.68) ^ab^	
Maxillary premolars	1.53	(0.94)	1.51	(0.74–2.15) ^b^	
Mandibular premolars	1.65	(1.11)	1.50	(0.86–2.28) ^ab^	
Maxillary molars	1.79	(1.33)	1.67	(0.73–2.65) ^ab^	
Mandibular molars	1.81	(1.29)	1.61	(0.77–2.79) ^ab^	

Note: Values are expressed as mean (SD) and median (IQR). Different superscript letters indicate statistically significant differences between tooth groups (Kruskal–Wallis test with Dunn–Bonferroni post hoc comparisons, adjusted *p* < 0.05). Tooth groups sharing the same letter do not differ significantly. * *p* < 0.05.

**Table 3:** Linear regression model on tooth-level resorption score as the dependent variable and age, gender, facial pattern, and tooth group as predictors.

Variable	Coefficient	Std. Error	t-Statistic	*p*-Value
(Intercept)	1.345	0.142	9.505	0.000 *
Age (years)	0.015	0.006	2.400	0.017 *
Gender: feminine	−0.050	0.076	−0.650	0.516
Facial pattern: hypodivergent	−0.041	0.075	−0.556	0.579
Facial pattern: hyperdivergent	−0.066	0.082	−0.807	0.420
Mandibular premolars	0.124	0.120	1.036	0.301
Maxillary molars	0.264	0.142	1.865	0.062
Mandibular molars	0.286	0.150	1.912	0.056
Maxillary canines	0.315	0.132	2.387	0.017 *
Mandibular canines	0.230	0.136	1.689	0.092
Maxillary central incisors	0.568	0.127	4.481	0.000 *
Mandibular central incisors	0.387	0.138	2.813	0.005 *
Maxillary lateral incisors	0.497	0.135	3.691	0.000 *
Mandibular lateral incisors	0.385	0.135	2.859	0.004 *
Model summary: R^2^ = 0.026; adjusted R^2^ = 0.017; F-statistic = 2.835, *p* < 0.001				

Note: Reference categories: male sex, normal facial pattern, and maxillary premolars as tooth group; * *p* < 0.05.

**Table 4:** Spearman correlations between age and mean external root resorption.

	Mean External Root Resorption				
	**General**	**Anterior-Superior**	**Posterior-Superior**	**Anterior-Inferior**	**Posterior-Inferior**
**Age**	0.135	0.034	0.108	0.250 *	−0.110

Note: * *p* < 0.05.

**Table 5:** Comparison of mean external root resorption (mm) by gender.

Mean External Root Resorption	Gender				*p*
	Male		Female		
General	1.72	(0.58)	1.73	(0.56)	0.935
Anterior-Superior	1.97	(1.00)	1.86	(0.78)	0.543
Posterior-Superior	1.58	(0.60)	1.49	(0.71)	0.539
Anterior-Inferior	1.57	(0.90)	1.79	(1.03)	0.368
Posterior-Inferior	1.43	(0.94)	1.57	(0.84)	0.499

Note: Statistical test used—Mann–Whitney U Test.

**Table 6:** Comparison of mean external root resorption (mm) by facial pattern.

Mean External Root Resorption	Facial Pattern						*p*
	Hypodivergent		Normodivergent		Hyperdivergent		
General	1.75	(0.53)	1.74	(0.58)	1.70	(0.60)	0.917
Anterior-Superior	1.92	(0.82)	1.86	(0.82)	1.85	(0.88)	0.925
Posterior-Superior	1.51	(0.69)	1.55	(0.69)	1.46	(0.68)	0.881
Anterior-Inferior ^†^	1.73	(1.04–2.52)	1.33	(0.91–2.22)	1.85	(1.10–2.40)	0.434
Posterior-Inferior	1.42	(0.78)	1.72	(0.93)	1.42	(0.81)	0.222

Note: Statistical test used—Kruskal–Wallis H Test, ^†^ Values presented as median and interquartile range.

**Table 7:** Comparison of mean external root resorption (mm) by type of dento-maxillary anomaly (Angle’s classification).

Mean External Root Resorption	Type of Dento-Maxillary Anomaly (Angle’s Classification)								*p*
	Class I		Class II/1		Class II/2		Class III		
General	1.78	(0.58)	1.75	(0.64)	1.67	(0.54)	1.71	(0.50)	0.868
Anterior-Superior	2.00	(0.77)	1.61	(0.71)	1.89	(0.96)	1.86	(0.82)	0.374
Posterior-Superior	1.46	(0.74)	1.65	(0.63)	1.50	(0.67)	1.55	(0.59)	0.837
Anterior-Inferior ^†^	1.86	(1.08–2.50)	1.67	(0.49–2.36)	1.20	(0.97–2.32)	1.78	(1.13–2.29)	0.643
Posterior-Inferior	1.59	(0.81)	1.52	(0.76)	1.52	(0.98)	1.48	(0.94)	0.976

Note: Statistical test used—Kruskal–Wallis H Test, ^†^ Values presented as median and interquartile range.

## Data Availability

All data are presented in the paper, further information will be provided upon request from the corresponding author.

## References

[1] Patel S., Ford T.P. (2007). Is the Resorption External or Internal?. Dent. Update.

[2] Thomas I. (2021). Review of Current Medical Literature on Root Resorption in Orthodontics. Braz. J. Implantol. Health Sci..

[3] Weltman B., Vig K.W.L., Fields H.W., Shanker S., Kaizar E.E. (2010). Root Resorption Associated with Orthodontic Tooth Movement: A Systematic Review. Am. J. Orthod. Dentofac. Orthop..

[4] Makedonas D., Lund H., Gröndahl K., Hansen K. (2012). Root Resorption Diagnosed with Cone Beam Computed Tomography after 6 Months of Orthodontic Treatment with Fixed Appliance and the Relation to Risk Factors. Angle Orthod..

[5] Dawood H.M., Kroeger A., Chavda V., Chapple I.L.C., Kebschull M. (2023). Under Pressure—Mechanisms and Risk Factors for Orthodontically Induced Inflammatory Root Resorption: A Systematic Review. Eur. J. Orthod..

[6] Picanço G.V., Freitas K.M.S.D., Cançado R.H., Valarelli F.P., Picanço P.R.B., Feijão C.P. (2013). Predisposing Factors to Severe External Root Resorption Associated to Orthodontic Treatment. Dent. Press J. Orthod..

[7] Albu Ş.D., Dragomirescu A.O., Albu C.C., Suciu I., Ionescu E. (2024). Genetic Polymorphisms of Interleukins IL-1A, IL-1B, and IL-1RN in Patients with Periodontal Disease and Dento-Maxillary Anomalies. Rom. J. Oral Rehabil..

[8] Heboyan A., Avetisyan A., Karobari M.I., Marya A., Khurshid Z., Rokaya D., Zafar M.S., Fernandes G.V.D.O. (2022). Tooth Root Resorption: A Review. Sci. Prog..

[9] Lopatiene K., Dumbravaite A. (2008). Risk Factors of Root Resorption after Orthodontic Treatment. Stomatologija.

[10] Pastro J.D.V., Nogueira A.C.A., Salvatore De Freitas K.M., Valarelli F.P., Cançado R.H., De Oliveira R.C.G., De Oliveira R.C.G. (2018). Factors Associated to Apical Root Resorption after Orthodontic Treatment. Open Dent. J..

[11] Nanekrungsan K., Patanaporn V., Janhom A., Korwanich N. (2012). External Apical Root Resorption in Maxillary Incisors in Orthodontic Patients: Associated Factors and Radiographic Evaluation. Imaging Sci. Dent..

[12] Fox N. (2005). Longer Orthodontic Treatment May Result in Greater External Apical Root Resorption: What Are the Treatment-Related Aetiological Factors of External Apical Root Resorption of the Maxillary Incisor?. Evid. Based Dent..

[13] Jung Y.-H., Cho B.-H. (2011). External Root Resorption after Orthodontic Treatment: A Study of Contributing Factors. Imaging Sci. Dent..

[14] Ionescu E. (2021). Anomaliile Dentare Și Dento-Maxilare.

[15] Linge L., Linge B.O. (1991). Patient Characteristics and Treatment Variables Associated with Apical Root Resorption during Orthodontic Treatment. Am. J. Orthod. Dentofac. Orthop..

[16] Brezniak N., Goren S., Zoizner R., Dinbar A., Arad A., Wasserstein A., Heller M. (2004). A Comparison of Three Methods to Accurately Measure Root Length. Angle Orthod..

[17] Lund H., Gröndahl K., Hansen K., Gröndahl H.-G. (2012). Apical Root Resorption during Orthodontic Treatment: A Prospective Study Using Cone Beam CT. Angle Orthod..

[18] Elhaddaoui R., Benyahia H., Azeroual M.-F., Zaoui F., Razine R., Bahije L. (2016). Resorption of Maxillary Incisors after Orthodontic Treatment–Clinical Study of Risk Factors. Int. Orthod..

[19] Sameshima G.T., Sinclair P.M. (2001). Predicting and Preventing Root Resorption: Part I. Diagnostic Factors. Am. J. Orthod. Dentofac. Orthop..

[20] Janson G.R.P., De Luca Canto G., Martins D.R., Henriques J.F.C., De Freitas M.R. (2000). A Radiographic Comparison of Apical Root Resorption after Orthodontic Treatment with 3 Different Fixed Appliance Techniques. Am. J. Orthod. Dentofac. Orthop..

[21] Tieu L.D., Saltaji H., Normando D., Flores-Mir C. (2014). Radiologically Determined Orthodontically Induced External Apical Root Resorption in Incisors after Non-Surgical Orthodontic Treatment of Class II Division 1 Malocclusion: A Systematic Review. Prog. Orthod..

[22] Pereira S.A., Corte-Real A., Melo A., Magalhães L., Lavado N., Santos J.M. (2024). Diagnostic Accuracy of Cone Beam Computed Tomography and Periapical Radiography for Detecting Apical Root Resorption in Retention Phase of Orthodontic Patients: A Cross-Sectional Study. J. Clin. Med..

[23] Merișescu M.-M., Luminos M.L., Pavelescu C., Jugulete G. (2023). Clinical Features and Outcomes of the Association of Co-Infections in Children with Laboratory-Confirmed Influenza during the 2022–2023 Season: A Romanian Perspective. Viruses.

[24] Dindaroglu F., Dogan S. (2017). Root Resorption in Orthodontics. Turk. J. Orthod..

[25] Luther F., Dominguez-Gonzalez S., Fayle S.A. (2005). Teamwork in Orthodontics: Limiting the Risks of Root Resorption. Br. Dent. J..

[26] Dumitrache M.A., Ionescu E., Sfeatcu R., Ginghina O. (2016). The Pharmacist’s Role in Preventive and Pharmaceutical Treatment for Oral Diseases. Farmacia.

[27] Komarina D., Hegde G., Shetty N.K. (2025). Root Resorption in Orthodontics: Revisiting The Evidence And Rethinking The Approach. IOSR J. Dent. Med. Sci..

[28] Wang J., Huang Y., Chen F., Li W. (2024). The Age-Related Effects on Orthodontic Tooth Movement and the Surrounding Periodontal Environment. Front. Physiol..

[29] Brezniak N., Wasserstein A. (2002). Orthodontically Induced Inflammatory Root Resorption. Part I: The Basic Science Aspects. Angle Orthod..

[30] Ciurla A., Szymańska J., Płachno B.J., Bogucka-Kocka A. (2021). Polymorphisms of Encoding Genes IL1RN and P2RX7 in Apical Root Resorption in Patients after Orthodontic Treatment. Int. J. Mol. Sci..

[31] Barroso M.C.F., Devita R.L., Lages E.J.P., Costa F.D.O., Drummond A.F., Pretti H., Lages E.M.B. (2012). Risk Variables of External Apical Root Resorption during Orthodontic Treatment. Dent. Press J. Orthod..

[32] Baghaei N.N., Zhai G., Lamani E. (2023). Genetic and Other Factors Contributing to External Apical Root Resorption in Orthodontic Patients. Orthod. Craniofac. Res..

[33] Kjaer I. (2013). External Root Resorption: Different Etiologies Explained from the Composition of the Human Root-Close Periodontal Membrane. Dent. Hypotheses.

[34] Bayir F., Bolat Gumus E. (2021). External Apical Root Resorption after Orthodontic Treatment: Incidence, Severity and Risk Factors. J. Dent. Res. Dent. Clin. Dent. Prospect..

[35] Sondeijker C.F.W., Lamberts A.A., Beckmann S.H., Kuitert R.B., Van Westing K., Persoon S., Kuijpers-Jagtman A.M. (2020). Development of a Clinical Practice Guideline for Orthodontically Induced External Apical Root Resorption. Eur. J. Orthod..

[36] Azeez S.M. (2023). Evaluating Diagnostic Performance of Three Cephalometric Vertical Parameters. Indian J. Dent. Res..

[37] Ahmed M., Shaikh A., Fida M. (2016). Diagnostic Performance of Various Cephalometric Parameters for the Assessment of Vertical Growth Pattern. Dent. Press J. Orthod..

[38] Rizwan M., Mascarenhas R., Husain A. (2011). Reliability of the Existing Vertical Dysplasia Indicators in Assessing a Definitive Growth Pattern. Rev. Latinoam. Ortodon. Odontop..

[39] Roy P., Roy P., Koley S. (2024). Comparative Assessment of Various Cephalometric Parameters Used for Determining Vertical Skeletal Dysplasia. Cureus.

[40] Taha M., Montasse M. (2012). Reliability of Cephalometric Measurements Utilized in Evaluation of the Vertical Facial Dimension. Egypt. Orthod. J..

[41] Özel M.B., Kartbak S.B.A., Çakmak M. (2025). Classification Performance of Deep Learning Models for the Assessment of Vertical Dimension on Lateral Cephalometric Radiographs. Diagnostics.

[42] Paranhos L.R., Torres F.C., Brando T.M., Kaieda A.K., Ramos A.L. (2014). The Inadequacy of the Y-Axis of Growth (SNGn) for the Vertical Pattern Assessment in Patients with Sagittal Discrepancies. J. Contemp. Dent. Pract..

[43] Saadeh M.E. (2025). Concordance of Cephalometric Classifications of Divergence in Orthodontics. Cureus.

[44] Fernandes L.Q.P., Figueiredo N.C., Montalvany Antonucci C.C., Lages E.M.B., Andrade I., Capelli Junior J. (2019). Predisposing Factors for External Apical Root Resorption Associated with Orthodontic Treatment. Korean J. Orthod..

[45] Scheibel P.C., Micheletti K.R., Ramos A.L. (2011). External Apical Root Resorption after Six and 12 Months of Non-Extraction Orthodontic Treatment. Dentistry.

[46] Krishnan V. (2005). Critical Issues Concerning Root Resorption: A Contemporary Review. World J. Orthod..

[47] Mavragani M. (2000). A Radiographic Comparison of Apical Root Resorption after Orthodontic Treatment with a Standard Edgewise and a Straight-Wire Edgewise Technique. Eur. J. Orthod..

[48] Macrì M., Medori S., Varvara G., Festa F. (2023). A Digital 3D Retrospective Study Evaluating the Efficacy of Root Control during Orthodontic Treatment with Clear Aligners. Appl. Sci..

[49] Sameshima G.T., Sinclair P.M. (2001). Predicting and Preventing Root Resorption: Part II. Treatment Factors. Am. J. Orthod. Dentofac. Orthop..

